# Supplementary material for: Butyrate Prevents TGF-β1-Induced Alveolar Myofibroblast Differentiation and Modulates Energy Metabolism
Source: Metabolites. 2021 Apr 22;11(5):258. doi: 10.3390/metabo11050258 (PMC8143476; doi:10.3390/metabo11050258)
Supplement: Supplementary file 1 [file metabolites-11-00258-s001.zip › metabolites-1161063-supplementary.pdf]

Butyrate prevents TGF-β1–induced alveolar myofibroblast differentiation and modulates energy metabolism

Hyo Yeong Lee [1,^], Somi Nam [2,^], Mi-Jeong Kim [2,^], Su Jung Kim [1], Sung Hoon Back [2,*], and Hyun Ju Yoo [1,*]

[1] Department of Convergence Medicine, Asan Institute for Life Sciences, Asan Medical Center, University of Ulsan College of Medicine, Seoul 05505, Republic of Korea

[2] School of Biological Sciences, University of Ulsan, Ulsan 44610, Republic of Korea

^ These authors contributed equally to this work.

Supplementary Figures

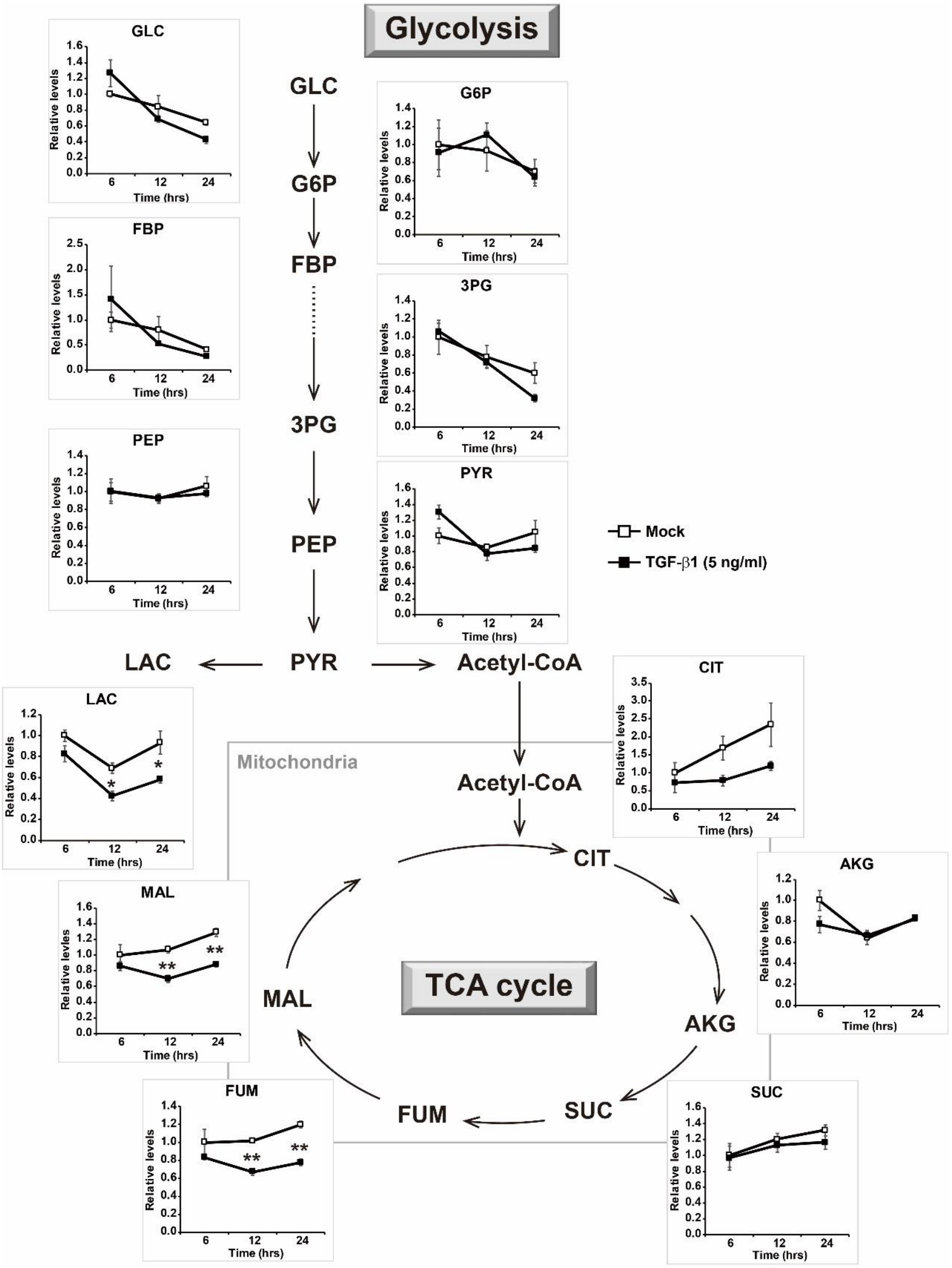

Figure S1. Effect of TGF-β1 on changes in metabolites involved in energy metabolism in pulmonary fibroblasts. Cells were treated with PBS (Mock) or 5 ng/ml TGF-β1 for 24 hrs. The data are expressed as means ± SEM of three independent experiments, *P<0.05 and **P<0.01; Mock-treated cells vs. TGF-β1-treated cells.

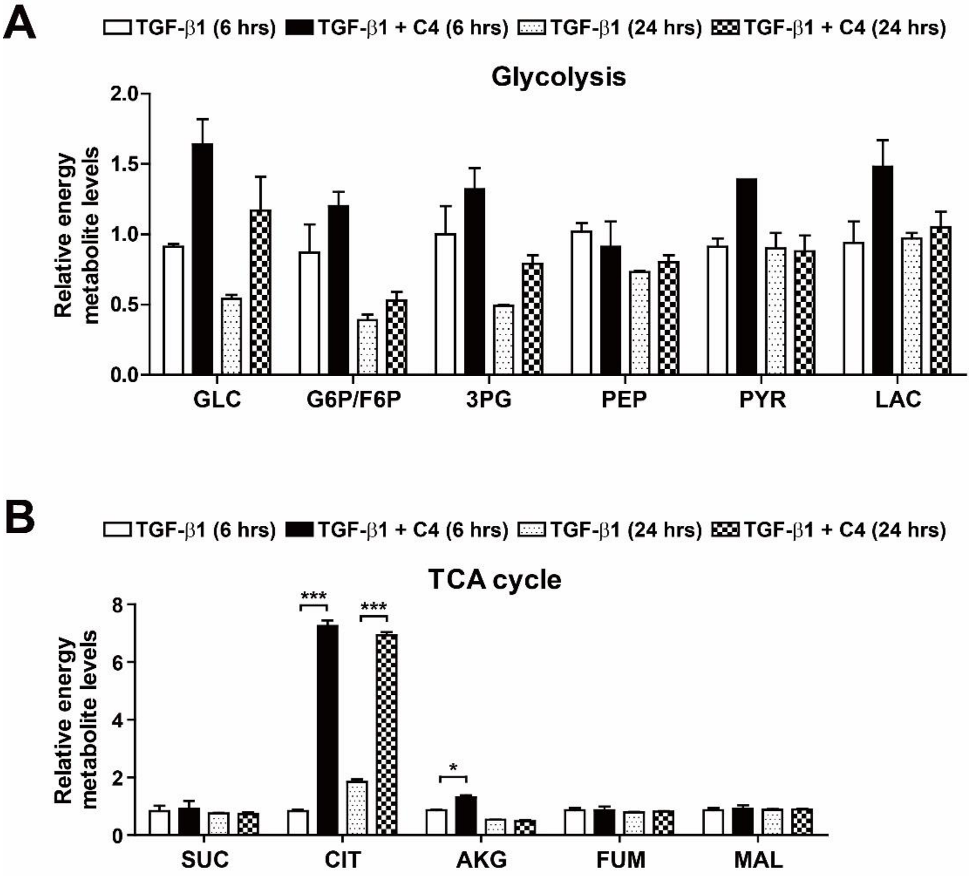

Figure S2. Effect of butyrate on changes in metabolites involved in energy metabolism in TGF-β1–treated pulmonary fibroblasts. MRC5 cells were treated with TGF-β1 (5 ng/ml) for the indicated times with or without C4 (5 mM). Data are expressed as means ± SEM of three independent experiments, *P<0.05 and ***P<0.001; TGF-β1–treated cells vs. TGF-β1 + C4-treated cells.

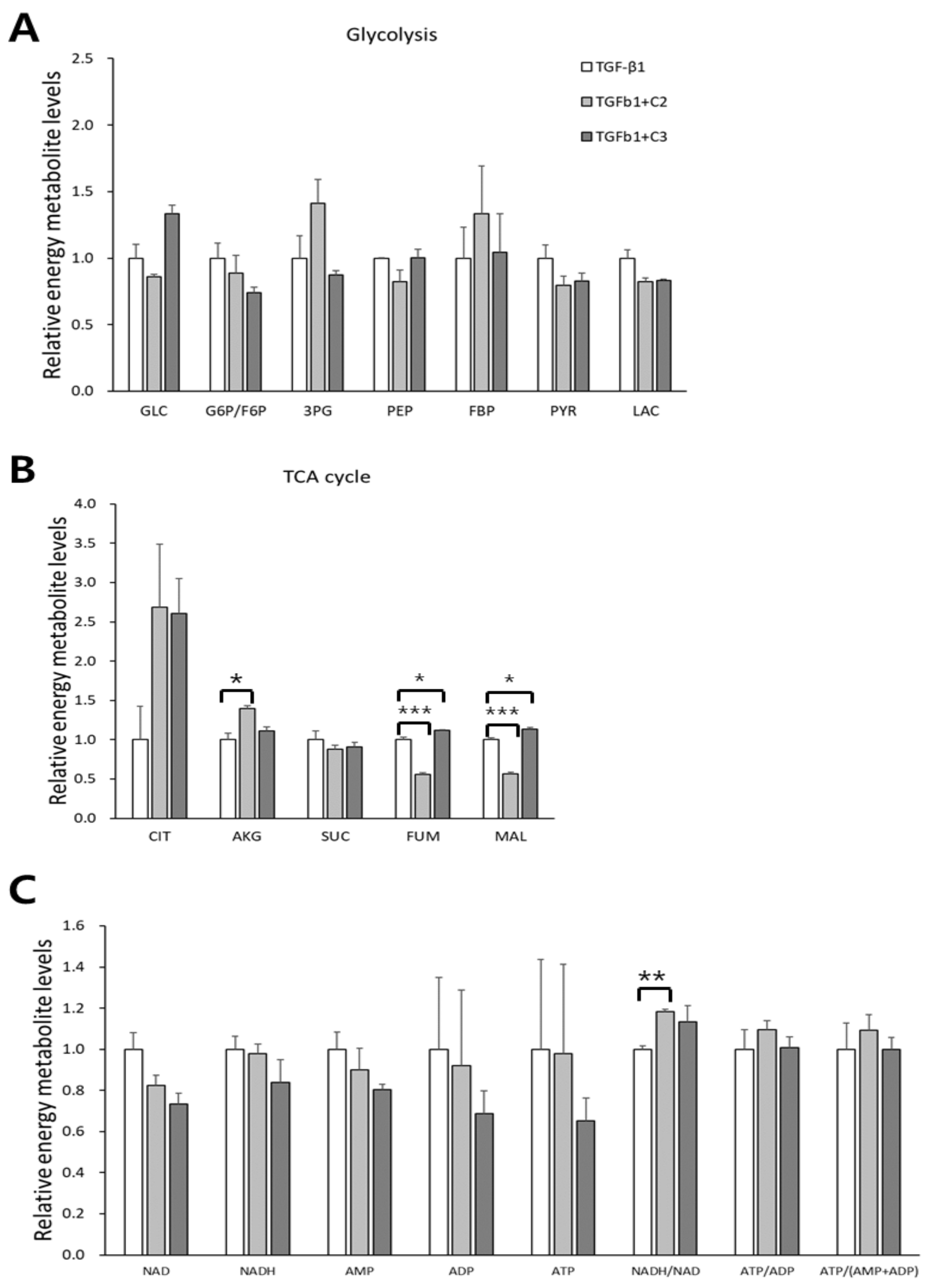

Figure S3. Effect of acetate and propionate on changes in metabolites involved in energy metabolism in TGF-β1–treated pulmonary fibroblasts. MRC5 cells were treated with TGF-β1 (5 ng/ml) for 24 hrs with or without C2 (5 mM) or C3 (5mM). Data are expressed as means ± SEM of three independent experiments, *P<0.05, **P<0.01and ***P<0.01; TGF-β1–treated cells vs. TGF-β1 + C2 (or C3)-treated cells.
